# Dimensions of Sexual Health Conversations among U.S. Black Heterosexual Couples

**DOI:** 10.3390/ijerph20010588

**Published:** 2022-12-29

**Authors:** Natalie M. Leblanc, Noelle M. St. Vil, Keosha T. Bond, Jason W. Mitchell, Adrian C. Juarez, Faith Lambert, Sadandaula R. Muheriwa, James McMahon

**Affiliations:** 1 School of Nursing, University of Rochester, Rochester, NY 14642, USA; 2 School of Social Work, University at Buffalo, Buffalo, NY 14215, USA; 3 School of Medicine, City University of New York, New York, NY 10031, USA; 4 Robert Stempel College of Public Health & Social Work, Florida International University, Miami, FL 33174, USA; 5 School of Nursing, University of Texas Medical Branch, Galveston, TX 77550, USA

**Keywords:** dyadic analysis, sexual health, black couples, heterosexual, health communication

## Abstract

Sexual health communication is an important feature of healthy intimate relationships; however, some couples may avoid discussing difficult matters (e.g., HIV/STI testing, sexual satisfaction) to minimize interpersonal conflict. From October 2018 to May 2019 in New York State, we conducted a multi-method descriptive pilot study to characterize Black heterosexual couples’ (N = 28) sexual health conversations. Partners individually completed an online sexual health/relationship survey before engaging in-person for a joint dyadic qualitative in-depth interview. Quantitative descriptive statistics demonstrated that most absolute score differences among couple’s preferences for sexual health outcomes, communal coping and sexual relationship power were mainly small, but greatest regarding extra-dyadic sexual behaviors. A qualitative descriptive approach discerned, motivation and norms for sexual health conversations, and communication patterns. Thematic and content analysis revealed two central themes: *initiating and sustaining sexual health conversations*, and *leveraging features of the couples to promote sexual health*. Integrated findings indicate that couples possess varied communication patterns that operate with motivations for sexual health conversations toward subsequent sexual health promotion. Equitable and skewed communication patterns emerged as relationship assets that can be leveraged to optimize sexual health. There is also opportunity for future work to address communication regarding extra-dyadic behavior and preferences. Asset-based considerations are discussed.

## 1. Introduction

Sexual health is not just the absence and treatment of disease and dysfunction, but also incorporates the physical, emotional, mental and social well-being of people and the intimate relationships they engage in [1]. However, the integration of sexual health and specifically sexually acquired diseases and infirmary with overall health and wellness remains a challenge to achieve [2]. This is in part due to siloes in research and public health that orients sexual health within a solely disease/morbidity-focused, deficit-based, or sexual dysfunction/satisfaction paradigm [2]. This paradigm, although warranted, may disallow other considerations such as the psycho-social attributes of sexual health, and subsequently the role of interpersonal relationships and the importance of interpersonal communication in sexual health promotion processes [3]. These considerations are warranted given that ongoing HIV/STI transmission is overwhelmingly sustained among couples and intimate partners, and subsequently within communities. Reports from the Center for Disease Control and Prevention [4] and the World Health Organization [5] indicate that up to 50% of heterosexual persons acquire HIV infection in relationships with partners who were confirmed to be living with HIV [6]. National statistics also demonstrate that problems with sexual functioning and satisfaction, which are more documented among males than female [7], affect up to 50% of the U.S. male population [8], is increasing among those in late adolescence and emerging adults [9], and may disproportionately impact males who are living with certain diseases such as HIV infection [7,10]. Sexual satisfaction is deemed indicative of relationship health, but can also be the result of undergirding health conditions or side effects from certain medications [1]. This context in sexual health requires that partners, especially in sustained and committed relationships, are able to communicate about such issues in order to maintain relationship health and integrity [11].

The inability to effectively communicate needs and desires is a source of conflict in many relationships [12]. However, an essential component of sexual health promotion is the communication, conversations, and subsequent mutual understandings and expectations that develop between intimate sexual partners [13]. Sexual health conversations among couples are important for disease prevention, to build intimacy, and establish norms pertaining to relationships preferences and desires. A 2018 meta-analysis on the effect of campaign-generated interpersonal communication on campaign-targeted health outcomes found that conversations with intimate partners such as a spouse, romantic partner, or significant other had the most impact on achieving health outcomes opposed to talking with others about these issues [14]. Such communication is also important to ensure that partners within a couple or other intimate sexual unions (i.e., polyamorous relationships, consensual non-monogamy) are able to develop a joint understanding of what sexual health promotion looks like for them. In order to successfully arrive at this place of joint understanding and shared decision making, the nature of sexual health communication is pivotal, specifically for marginalized groups experiencing disproportionate rates of HIV/STIs.

U.S. Black persons are disproportionately affected by adverse sexual health outcomes such as HIV/STI acquisition and delayed or lack of access to HIV/STI care. Although Black persons are more likely to undergo HIV/STI screening [15,16] and report less “riskier” sexual behaviors than other ethno-racial groups across their lifespan, they have a greater lifetime susceptibility to HIV infection and other STIs [16]. Threats to sexual health for Black persons and couples in particular are in large part due to historical–social structure and community-level vulnerabilities [17,18]. These vulnerabilities are fueled by anti-Black racism and discriminatory social policies and conditions (i.e., mass incarceration, high un/under-employment, low social capital, high community viral loads) combined with interpersonal factors (i.e., intimate partner violence, serial non-consensual non-monogamy) [19,20,21,22,23]. These threats to sexual health have been documented as contexts, which heighten Black persons’ susceptibilities to HIV/STI acquisition and other adverse sexual health outcomes [17,24,25].

Due to intersectional stigmas such as living with HIV infection, HIV/STI susceptibility, poverty, and experiences of anti-Black stigma, some couples may avoid conversations about sensitive topics (i.e., HIV/STI testing, sexual pleasure) out of fear of embarrassment, relationship discord, or HIV/STI stigma [26]. Others may lack the necessary communication skills and desire assistance in facilitating sexual health conversations [27,28]. Sex and power dynamics also heighten HIV susceptibility among some Black female persons due to internalizing gender scripts to maintain or initiate relationships. This diminishes autonomy to communicate preferences regarding sexual safety and relationship goals to partners [22]. Furthermore, heterosexually acquired HIV infection among Black cisgender females is also attributable to interpersonal contexts including male cisgender partners being unaware of their HIV serostatus and engaging in concurrent sexual relationships [18,29,30]. In juxtaposition, Black couples are more likely to have non-conventional gender roles due in part to the anti-Black racism and discriminatory social policies noted above, which have resulted in Black female cisgender persons to have a higher levels of education and subsequent employment (though this does not translate to higher levels of wealth/income) [18]. This context may engender a feeling of resentment by both partners, further resulting in relationship discord and tension [18].

Given these contexts, discerning the motivations of current sexual health promotion behaviors, as well as the context and content of sexual health conversations among Black cisgender persons and their cisgender partners, is essential for addressing health disparities in sexual health, disease prevention, and relationship health. Such insights can also identify assets within couples in navigating their sexual health via communication, which can then be leveraged toward general health promotion. In this current report, we conducted a descriptive multi-method project from October 2018 to May 2019 to characterize and contextualize Black heterosexually identified, cisgender couples’ sexual health conversations. Couples were given the central prompt to: “Describe the sexual health conversations you and your partner have had before today.” From this prompt, couples engaged in a joint dyadic interview to describe and contextualize their conversations, practices, and behaviors regarding sexual health.

## 2. Materials and Methods

All procedures for the study and subsequent analyses were approved by [blinded] IRB. Our overarching objective for this analysis was to describe and contextualize the sexual health conversations and subsequent health considerations of Black-identified, self-defined cisgender couples residing in New York State. The purpose was to ascertain what are the conversations Black couples have as it pertains to sexual health and disease prevention, and what are the subsequent behaviors employed as a result or in tandem to these conversations. Such an inquiry identifies assets among couples that promote health, as well provide insights for intervention development to enhance sexual health communication.

We enrolled a purposive sample [31] of 28 Black-identified, self-defined heterosexual cisgender couples from 3 New York State jurisdictions using active and passive recruitment strategies. These strategies included posting flyers at community-based organizations, university research listservs and study information on social media. Subjects were encouraged via these strategies to either contact the study team directly and/or complete a REDCAP survey to assess eligibility (at least 18 years old and older, sexually active, Black-identified, in a relationship with someone not of the same sex, and partners mutually endorsed each other as the intimate partner). Details of study enrolment have been described elsewhere [32], and are briefly reported here. An index partner contacted the study team and was requested to provide contact information for themselves and their partner. Partners were separately contacted and prompted to confirm eligibility and couple’s verification [33,34] form also via REDCAP. Once eligibility and couple verification was established, each partner were individually sent a REDCAP link to consent to the study and asked to complete an online quantitative sexual health communication survey. The survey captured individuals’ preferences for sexual health outcomes, communal communication strategies and relationship power.

Once each partner completed the sexual health survey, they were then invited to an in-person, joint dyadic in-depth interview. Each participant was paid USD 50, and each couple was provided up to USD 12 for transportation. Quantitative descriptive statistics characterized preferences for sexual health outcomes, communal communication strategies, and relationship power. A qualitative descriptive approach [35] was used to characterize self-identified Black-heterosexual couples’ sexual health conversations. Joint dyadic interviews [36,37] identified joint perspectives regarding overall/sexual health promotion, motivation and norms for sexual health conversations, communication patterns, and relevant themes.

### Data Analysis

Quantitative descriptive statistics was conducted in SPSS and involved characterizing the sample’s individual sexual history including HIV/STI screening, history of HIV/STI acquisition and treatment. Descriptive statistics also assessed relationship dynamics using selected subscales from HIV-Related Dyadic Measures [38] and the Sexual Relationships Power Scale [39] with all responses measured on a Likert Scale. In this study, to be mindful of participant burden, not all constructs from the full HIV-Related Dyadic Measure were used. The HIV-related dyadic measure included the following constructs which contained the exemplar activity or perspective statement in parentheses: *couples’ preferences for sexual health outcomes (e.g., Using condoms with your current partner)* prompted participants to indicate to what extent they viewed this as their personal decision versus a joint decision on a Likert Scale of 1 = ‘your issue/decision’ to 5 = ‘our issue/decision’; and *communal coping on communication to reduce HIV threats (e.g., Being sexually faithful to each other)* in which participants had to indicate to what extent they communicated about the topic on a Likert Scale of 1 = ‘Not to any extent at all’ to 5 = ‘To a great extent’. The sexual relationship power scale included 2 subscales/constructs of relationship control and decision-making dominance [39]. The first construct *(relationship control)* contained 15 items that, prompted participants with a statement (e.g., *Most of the time, we do what my partner wants to do)* followed by, a response that best described their perception of the relationship with their partner using a Likert Scale of 1 = ‘Strongly disagree’ to 4 = ‘Strongly agree’. The second construct (*decision-making dominance)* contained 3 items that prompted participants to respond to a question (*e.g., in general, who do you think has more power in your relationship?)* with categorical responses that they perceived best characterized themselves and their partner using 1 = ‘Your partner’, 2 = ‘Both of you equally’, and 3 = ‘You’.

Responses from the HIV-Related Dyadic Measures of *couples’ preferences for sexual health outcomes and communal coping on communication to reduce HIV threats* [38] and the Sexual Relationships Power subscale, *relationship control* [39], allowed for the absolute differences between partners to be calculated by determining the difference between individual responses. Absolute score differences (i.e., average difference) and couple-level means were calculated for responses to Likert-type scales and items. Average absolute score difference was only calculated for Sexual Relationships Power subscale relationship control, which had a response option range pertaining to agreement about the statement (i.e., strongly dis/agree). The absolute score difference measured the extent of concurrency between partners’ responses regarding preferences for sexual health outcomes and whether these preferences were individual or a shared decision. A low absolute score difference indicated that couples had concurrent or complimentary responses to the constructs and specific questions. It essentially demonstrated whether they believed certain decisions were a joint decision or their personal decision. A greater absolute score difference demonstrated that the couples had disparate views or preferences to the construct or a specific topic. The construct, decision making dominance, did not have absolute score differences to calculate.

A qualitative descriptive design [35] characterizes the approach and analysis of the joint interviews. Atlas.ti was used as the data management tool to assist with the qualitative analysis. The qualitative dyadic analysis simultaneously entailed two components [36,37,40]. One component was an assessment of the couple’s interaction. This not only entailed what was being said between partners, but the interaction and attributes of the couples in how they conveyed their experience in cultivating sexual and relationship health conversations and communication in their relationship. Therefore, the dyadic qualitative data analysis comprised two dimensions. The first entailed the perceived interpersonal dynamic as demonstrated by observing the in-person interviews, assessing the language used and interchange between the partners, reading/re-reading the transcripts to assess the communication patterns as they showed up on the page and peer debriefings. This analysis also gave insight to relationship dynamics that was demonstrated either when couples discussed a particular topic and/or during the overall interview. Dynamics also entailed a consideration of the sex of the partner and the age difference between partners. The second component of the qualitative dyadic analysis entailed the interview content utilizing a descriptive qualitative approach. A thematic analysis involved an inductive iterative approach in the generation of codes and then (sub)themes. This approach identified joint perspectives regarding overall/sexual health promotion, motivation and norms for sexual health conversations, communication patterns and relevant themes. A peer debriefing consisting of 3 co-authors and a consultant was conducted to ensure that there was agreement with the findings, coding and thematic scheme.

Qualitative analysis resulted in the emergence of two reflective themes from couples reports and narratives regarding sexual health conversations prior to participating in the current study. The first theme is dimensions of sexual health conversations; and the second theme is leveraging features of couples to promote sexual health promotion. Sexual health conversations are those by which the couples report issues pertaining to disease prevention, sexual functioning, establishing of preferences, intimacy building and related behavior, and, more broadly, relationship health.

## 3. Results

### 3.1. Study Sample Characteristics

Study sample characteristics have been reported in detail elsewhere [32]. As noted in Table 1, all partners resided in the same city and most of the 28 couples resided in Western New York State (*n* = 21), whereas the remaining were from New York City. The mean age of couples was 43 years old, and the average age difference between partners was 6.7 years. Most couples were married, engaged or dating and mutually endorsed their relationship type and length. Couples’ relationship length varied with most reporting (*n* = 18) being together for longer than 5 years. At the couple level, many of the relationships in the sample had both partners share the same characteristic or reported the same information about their demographic, including ethno-racial identity, metro area of residence, current sexual identity, and cohabitation. Instances in which both partners of the couple did not concur or reported not having the same characteristic included educational attainment, employment status, and, to a lesser degree, their relationship type and relationship length. A lack of concurrence meant, for example, that one partner may be working part-time and the person is a student, or that one partner has a college degree and the other an associate’s degree. In our assessment across the sample we did not find that any differences of these characteristics between partners had bearing on sexual health behaviors or relationship dynamics.

#### 3.1.1. Couples’ Sexual Behavior

About two-thirds of individuals reported engaging in vaginal intercourse and oral sex (62.5%, *n* = 35), whereas 29% (*n* = 16) noted they only had intercourse with their relationship partner in the last 6 months; fewer reported oral sex only (2%, *n* = 1) or oral, vaginal, and anal sex (7%, *n* = 4) with their current partner. The average number of times individuals had intercourse in the past 6 months was 9.22 (SD = 10.08). Frequency of oral sex within the last 6 months ranged between none to 30 times, and one individual noted having had anal sex once during the same time frame. For all types of sex, condoms were rarely used; 4 couples reported consistent condom use for vaginal intercourse.

Relative to HIV prevention and treatment, 25% (*n* = 14) of participants reported never having tested for STIs, whereas 66% (*n* = 37) had ever, and 9% (*n* = 5) refused to provide a response. Among those who had been tested, the last STI test occurred approximately 3.3 years prior (SD = 6.00). Almost two-thirds (65%, *n* = 31) noted that they have had an STI in their lifetime. In total, 75% (*n* = 42) of participants reported that they have been tested for HIV in the past, 18% (*n* = 10) self-reported never having tested, and 7% (*n* = 4) refused to provide a response. Among those who had been tested, their last HIV test occurred approximately 3.7 years ago (SD = 6.72). Eleven participants (*n* = 23%) self-reported living with HIV, and three refused to provide a response. Overall, 14% (*n* = 8) of participants had never been tested for STIs and HIV compared with 61% (*n* = 34) who have been tested for both.

At the couple-level, 19 couples reported being sero-concordant HIV-negative, five as HIV sero-discordant, and three as sero-concordant HIV-positive. Both partners of one couple refused to provide answers to questions about their sexual health and behaviors in the survey; their couple HIV serostatus was denoted as unknown. All participants living with HIV reported they had been linked to HIV care, that all partners knew their HIV status, and all but one was currently on antiretrovirals (ART).

#### 3.1.2. Relationship Dynamics

In this study, quantitatively we were exclusively interested in communal preferences for sexual health outcomes, specifically: communal coping on communication, and perceived relationship power. Table 2 shows the mean responses to relationship dynamic constructs across the sample of individuals, and average absolute score differences are reported. Not shown are sex-based scores, in which we were able to determine that there were no distinct trends by sex. Regarding preferences for sexual health outcomes, on average, couples’ absolute score differences were the highest about: having sex with someone other than the main partner (M = 1.89, SD = 1.75); whether condoms will be used when having sex with someone other than the main partner (M = 1.43, SD = 1.84); getting an STI or HIV test (M = 1.36, SD = 1.55). On average, couples’ absolute score differences were the lowest when it pertained to HIV/STI prevention (M = 1.18, SD = 1.45) and condom use with the study-involved partner (M = 0.86, SD = 1.23).

The absolute score difference revealed whether couples concurred on their ability to communicate about using HIV prevention strategies (i.e., communal coping). In this regard, couples’ absolute score differences were the highest, on average, when it pertained to communication about decisions related to extra-dyadic sexual activity: having sex outside of the relationship (M = 2.36, SD = 1.60); limiting the number of other sex partners (M = 2.04, SD = 1.54); using condoms when having sex outside of the relationship (M = 1.71, SD = 1.64). On average, couples’ absolute score differences were the lowest when it pertained to them communicating about decisions related to: using condoms in their relationship (i.e., with each other; M = 1.25, SD = 1.42); being sexually faithful to each other (M = 0.79, SD = 0.95); getting tested regularly for STDs and/or HIV (M = 1.25, SD = 1.37).

The absolute score difference in relationship control on the sexual relationship power scale indicated that on average, participants largely disagreed that their partner exhibits greater control about sexual health in their relationship. Given the response options for the decision-making dominance construct, no absolute score difference was calculated. Not shown in Table 2 is that most couples (*n* = 20) had both partners with an average score of 2 [range: 1.67–2.33], which indicated congruence in perceiving that there is absolute or mainly equality in decision making in the relationship. The remaining couples (n = 6) demonstrated a slightly skewed score toward female dominance in which the female partners averaged a score 2 or greater and the male partners in these couples having an average score of less than 2. The last two couples demonstrated some skewedness toward male dominance.

### 3.2. Qualitative Findings Themes, Sub-Themes and Dimensions

The qualitative dyadic analysis entailed not only what is being said, but also the interaction and attributes of the couples in how they conveyed their experience in cultivating relationships and sexual health communication. It entailed whether couple’s joint dialogue and contribution to the interview were open and balanced, with partners having an equal say on a particular issue or throughout the interview. It also entailed partners’ engagement with each other during the interview, and whether they demonstrated a harmonious egalitarian manner and/or were there certain conversations and topics that engendered tension or discord. Observational dynamics and quantitative descriptive data also considered the partner sex and age differences. Overall, there was concurrency in demographic factors between partners. In couples where the female partner was older (*n* = 11), there was a tendency for there to be more conversation during the interviews; however, quantitative findings did not differ based on these characteristics.

#### 3.2.1. Dyadic Observations of Couple’s Communication Patterns

Most couples who exhibited an equitable power dynamic (*n* = 18) had an open communication pattern during the interviews, and conveyed a more harmonious disposition throughout the interview. They also reported ongoing sexual health conversations throughout their relationship. Other couples (*n* = 7 couples) had a skewed dynamic in which one partner was more vocal at times or throughout the interview; nevertheless, the other partner remained engaged. Such couples endorsed intermittent sexual health conversations to address particular issues as they arose, but these conversations may also be ongoing. Couples who demonstrated equitable and skewed communication patterns were motivated by seeking relationship transparency and approached shared decision making as a practice. In a few cases (*n* = 2 couples), one partner emitted a domineering presence at key points or during the entire interview, with the other partner minimally engaged either throughout the interview or at certain points of discussion. Communication patterns in this regard held more tension than the others and usually revolved around a particular issue (e.g., ethno-racial identity as a factor in one’s sexuality/sexual expression, unresolved sexual dysfunction issues, and infidelity).

Two reflective thematic categories were developed from couples reports regarding sexual health conversations prior to participating in the current study: *Dimensions of sexual health conversations*, and *Leveraging sexual health conversations toward health promotion behaviors*. Within each theme, subthemes and dimensions were developed to further characterize sexual health communication among couples.

#### 3.2.2. Theme 1: Dimensions of Sexual Health Conversations

**Figure 1:**
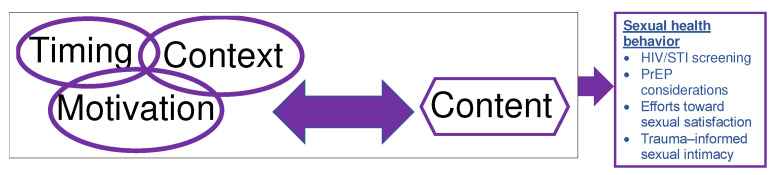
Attributes of sexual health conversations that influence the consideration and adoption of sexual health behaviors.

Couples’ reports on initiating and sustaining sexual health conversations entailed communication specific to relationship intention, sexual practices, efforts and challenges toward building intimacy, and establishing relationship norms. This theme is characterized by three attributes of these conversations: *timing of sexual health conversations, motivation for sexual health conversations,* and the *context of sexual health conversations*. These attributes were conceptualized as being co-related and interdependent, and operated synergistically to frame couple’s sexual health conversations. These attributes together are influenced by and influences the content of sexual health conversations among couples in this study. The content of conversation and the synergy of timing, context and motivation further initiates sexual health related behaviors that enhances or threatens sexual health and, in some instances, more broadly relationship health (Figure 1).

Sub-theme: Timing of Sexual Health Conversations

Couple’s described when sexual health conversations occurred in their partnership. For some couples, sexual health conversations commenced as a way of establishing norms and determining intention or stating expectations for a relationship. These conversations were initiated before a relationship commenced and to set intentions. Other conversations sought to reinforce or re-visit relationship expectations once the relationship had commenced. One dimension of timing was the frequency of conversations, including the spontaneity or longitudinal nature of certain conversations, which were at times due to certain topics (i.e., HIV/STI screening, dispensing of condom use). Timing attributes of sexual health conversations may also involve an in-the-moment experience the couple was having or conversations that were ongoing due to partners’ personalities or habits of speaking to each other about health topics more broadly as routine practice. In the latter instance, having conversations about sex-based subject matter was deemed normative in the relationship—“we talk about this stuff all the time”.

*M: …we asked each other if we’ve had partners before. How many partners? did we use protection? did we get tested? and we shared that. We didn’t have a problem with that…*

*F: …we just recently had a recent conversation…We thoroughly discussed our sexual history with each other. Like things that we–*

*M: Oh yeah, yeah. Yeah.*

*F: …things that we might have not told each other before…*

*M: Yeah, we talked about it and who those partners were*

*F: …Because there were people that I thought you did not have sex with, and you actually told me that you did.*

*M: One person!*

*F: I’m not mad at you…I’m just saying we had the conversation…*

HIV-concordant-negative couple (>5 yrs.)

Older couples in particular, who tended to be together longer (>5–10 years), noted that due to their relationship longevity, communication was more open, and ongoing. Another dimension to the timing of sexual health conversations was regarding the anticipation of a particular occurrence or experience due to the sexual history of one or between partners, and the perceived need for certain topics to be continually revisited. For example, a couple in which one or both partners are living with HIV infection or one partner has a history of sexual victimization. In these instances, couples reported checking in or re-affirming sexual (or intimate) behaviors, desires, and intention. Sexual health conversations in the latter instance included non-victimized partners seeking permission to commence intimate touching and to ensure their partner’s comfort and feelings of safety in their relationship. This relates to the timing of sexual health conversations; in particular, the dimension of intentionality is the motivation for sexual health conversations.

Sub-theme: Motivation for Sexual Health Conversations

The sources of motivation to engage in sexual health conversations are characterized by couples reports on the impetus of discussions on certain issues. The source of motivation comprised two dimensions: intrinsic motivation and extrinsic motivation. Certain conversations were due to intrinsic attributes of one or both partners wanting to communicate certain sexual and relationship preferences in the current relationship. It entailed a desire to engage in behaviors that support sexual and relationship expectations and norms, to establish new boundaries due to evolving priorities, or renewing preferences in the current relationship. These expectations and norms could be those that have been established for themselves from previous relationships. For example, couples reported on past experiences such as infidelity with previous partners or successful attributes and behaviors they now want considered and introduced in the current relationship.

*F: …It was me saying, listen, this is what we’re not going to do…it was an actual situation that we went through…if he did give me permission to go sleep around…I’m not going to go do it because it’s just not my thing…*

HIV-concordant-negative couple, (<5 yrs.)

*M: …we met at our primary care place…we were open beings, with like the questions, …‘do you know your status?’ You know different-or asking like ‘are you looking for a monogamous relationship?’ You know or ‘do you want an open-relationship?’…sometimes you have to ask those questions to know if you even want to go there because I like being with one person…*

HIV-concordant-negative couple, (>5 yrs.)

*F: We just recently had one [conversation]…A couple weeks ago.*

*M: We’ve been [together] for over twenty-two years…We talk about private ways to spice up our sex life…and what we’re capable of doing and what we aren’t capable of doing based on our age and our physical health.*

*F: The body changes. All types of things…It just happens.*

HIV-concordant-negative couple, (>20 yrs.)

Extrinsic motivation is that which is externally sourced from the individual self and influenced by observations of other people’s experiences, especially that related to adverse health and relationship outcomes. Individual partners and some couples reported avoiding adverse outcomes as the motivation for incorporating sexual health promotion practices such as routine (annual) HIV screening, relationship fidelity, and an open communication ethic as normative practice.

*M: …all my friends caught some type of STD…even like finding out that you could catch certain things just by oral sex…I just don’t never want to catch anything, even if I can get rid of it…And I never have…*

*F: That’s good. Just because you ask somebody something don’t mean you going to get the truth.*

*M: That’s true. But I still ask them…there’s no excuse…even if you feel like you’re not exactly confident with the person telling you, just either don’t move forward or you use condoms and you live with your decision…*

HIV-concordant-negative couple, (>5 yrs.)

Sub-theme: Context of Sexual Health Conversations

Couples reports on the context of their conversations entailed the circumstances surrounding “the why” these conversations occur or the occurrence which precipitated these conversations in the first place. Context revealed what was happening in the relationship or the experiences the couple had or was undergoing that warranted communication. The contexts of these conversations included disease prevention and treatment, either while living with HIV infection and preventing couple’s transmission (e.g., dispensing condoms, or incorporating pre-exposure prophylaxis for HIV prevention [PrEP]) or experiencing pregnancy. Women who experienced pregnancy were routinely screened for HIV infection, and it is during this period that female partners learned their HIV status. This subsequently initiated a conversation with male partners about HIV/STI screening. In other cases, male partners reported during the context of courtship, initiating conversations about HIV/STI screening and sex partner history while seeking relationship intention. Contexts also included revisiting relationship expectations due to breaches in expectations caused by infidelity.

*F: …we will have discussions because he is [HIV] positive…we constantly go to the doctor every three months…we find out what his viral levels are, and I’m still negative, and he still looks to me, ‘do you still want to be in this relationship?…*

*M: …that’s just the insecurity in me. That’s all.*

*F: …So five years later it’s still discussed. We still have those questions.*

*M: …Because you got to make sure…anybody that has this develops some type of complex…she’ll say, that girl’s all up in your face right and I be like okay, so what…why would I risk that?*

*F: …you may not look it and she might be open minded and…have everything you need that I don’t have. That’s the discussion that we still have with each other. Like, I’m not going to leave you, you might just find a broad that’s better than me. I have to worry about that…is my love still there, am I still your number one. You know we’re both saying to each other, am I still your number one.*

Serodiscordant couple, Male HIV+, (5 yrs.)

Other contexts of sexual health conversations occurred when couples were either dealing with unresolved sexual dysfunction problems, or seeking strategies to increase sexual satisfaction. Other conversations were related to maintaining an active sex life while considering physical adaption to an aging body. Couples reports on aging and sexual health were not just regarding evolving physical limitations, but included the side effects of medication for metabolic conditions that effect sexual functioning including HIV treatment, diabetes or psychiatric medications. One female partner during the course of the interview learned for the first time that her male partner was using Viagra as a way of mitigating the sex-based side effects of other medications. Younger couples (<35 years old,) did not necessarily report on sexual functioning issues, but more to concerns or discussions regarding relationship sustainability and health. Some female partners specifically spoke to the intended longevity of their relationships in tandem with fidelity and monogamous expectations.

#### 3.2.3. Theme 2: Leveraging Sexual Health Conversations toward Health Promotion Behaviors

Couples implicitly and explicitly reported on sexual and relationship health conversations, as well as attributes of their relationships or experiences that allows them to work towards sexual health promotion strategies. Embodying this theme are two subthemes: conversations and adoption of sexual health behaviors, and conceptualizing overall health-promotion goals.

Sub-theme: Conversations and Adoption of Sexual Health Behaviors

Couples reported on sexual health behaviors and practices that they have developed because of the sexual health conversations they had together. Considerations regarding for example, PrEP uptake for HIV prevention is a sexual-health-promoting activity Conversations regarding PrEP primarily occurred within the context of HIV serodiscordant couples in which partners were mutually aware of their HIV status, and all partners who discussed living with HIV in the interviews were on anti-retrovirals (ARTs). Among these couples, not a single person reported currently being on PrEP. In a few cases, partners initiated PrEP and discontinued because they did not like the side effects or no longer felt the need to use it. In one case, the female partner was on PrEP for conception purposes because her partner is living with HIV, and then discontinued its use. Although no one was currently taking PrEP, among those who reported using it, the sexual-health-promoting behavior is that the intervention was discussed, and a decision was made in the context of individual partners’ experiences and couples’ goals.

*F: …it was the effects of PrEP, I didn’t like them—the bone loss. And I’ve already had a number of accidents with bone loss…I am in a relationship with someone who has HIV, but I said there’s gotta be something else…to use as a safe-ty measure, back-up…I mean look what it’s doing to my bones..*

*M: Especially now, I’ve been on my HIV meds for quite a while. And I’m undetectable. And to my knowledge…I can’t infect anybody. Yeah.*

*F: …initially we discussed going on it…Just the same way coming off it.*

Serodiscordant couple, Male HIV+, (>5 yrs.)

Some partners reported a pattern of repeated or periodic HIV screening and sharing of HIV testing results as normative behavior. One female partner revealed the feelings of betrayal and embarrassment while receiving a STI diagnosis due to the current male partner’s infidelity. To reconcile these feelings of distrust and relationship tension, there was a joint decision that the male partner adopted routinized HIV screening as a HIV/STI prevention intervention for the couple. HIV screening of the female partner may be the first time anyone is being tested in the current relationship. In some instances, although the female partner had screened for HIV infection, the male partners did not report immediately screening even in the case whereby HIV infection was detected in the female partner. Despite normalization of routine HIV screening, less discussion included the consequences if one of the partners learned with their latest test that they were diagnosed with HIV or another STI.

Sexual health conversations also included communicating about intimate touching, comfort, physical intimacy, and vocalizing of behaviors that were off limits. For example, one couple described their engagement in intimate behaviors (not sexual intercourse) and navigating what sexual intimacy entails for them given the female partner experiences as a survivor of childhood sexual assault. In being attentive to her lived experience, the male partner reported being cognizant of his partner’s verbal and physical reactions to his engagement with her. This required him to be more vocal of his desires, and seeking permission to engage in provocative touching. Another couple described how they considered and acted on ways to expand their sexual repertoire, and arrived to a consensus whereby they realized that certain behaviors (i.e., anal sex) were practices they did not want to include. This couple reported freely engaging in ongoing conversation about sexual satisfaction, particularly given that they are first-time parents which has impacted their time and energy and this conversation further involved to what expanding their repertoire as couple could mean for them. Couples acknowledged the difficulty of ongoing conversations of this nature, but were motivated in that it has resulted in enhanced conversations and has built intimacy in their relationships.

*F: …I’ve experienced sexual abuse…*

*M: …that’s a hard conversation to have…the previous experiences that weren’t by choice…*

*F: …that’s’ something that my partner would have to be aware with-he can’t do certain things…*

*M: …ask permission before doing things that someone who hasn’t been abused would consider normal…do you mind me sharing…?*

*F: Yeah ((Laughing))*

*M: So like for instance, if I were to caress her breast, it causes her to tense…*

*F: Yeah, be on defense.*

*M: So now I ask permission, like is it okay if I touch your breast?…like figure out a way to ask permission or like what are your triggers and then be sensitive to those.*

HIV-concordant-negative couple, (<5 yrs.)

Sub-theme: Conceptualizing Overall Health Promotion Goals

Beyond sexual health conversations, couples reported on conversations they had related to general health promoting efforts for themselves, as a couple and as a family. The content of these conversations ranged from healthy eating, addressing a new health diagnosis, increasing physical activity, and smoking cessation. These conversations resonated across all couples, and involved being mindful of incorporating a healthy lifestyle into their relationships. This not only pertained to the physical self, but also social and interpersonal considerations that included thinking and re-considering their social network as a couple.

*M: I try to keep her health up like with black sea oil…I try to keep my fitness as well.*

*F: …he loves to eat healthy…because of the situation that he’s in.*

*M: [And I like to cook]…that’s one of the things like we developed in this relationship…I try to keep her from smoking cigarettes…But I can’t say too much because I smoke pot…we discuss a lot of stuff…*

*F: …like trying to have life insurance for our family…*

Serodiscordant couple, Male HIV+, (5 yrs.)

For some of the older couples (together for > 5–10 years, >45 years old), conversations entailed efforts towards health promotion due to emerging health concerns from one or both partners such as a family member’s new diagnoses of diabetes and couples subsequently discussing the need for or actually engaging in life-style changes. There were no differences between those couples living with HIV and those who were not when it came to overall health promotion. However, HIV infection and being on antiretrovirals with self-reported viral suppression, appeared to allow those particular couples to have conversations about overall health. This reality was factored into promoting overall health for themselves, their partners, and their families.

## 4. Discussion

Sexual health conversations are an integral component of healthy relationships including disease mitigation within partnerships. This study described the dimensions, context and content of sexual health conversations, as well as relationship characteristics and health behaviors among Black-identified, self-defined, heterosexual cis-gender couples residing in New York State. Two reflective thematic categories emerged from couples’ joint dyadic interviews: dimensions of sexual health conversations, and leveraging sexual health conversations toward health-promotion behaviors. In our study, couples’ dimensions of sexual health conversations embodied three intersecting attributes: timing, motivation, and context. The dimensions of these attributes were important to amplify because they spoke to the diversity of couples’ experiences within varying contexts. They also demonstrate the nuances that exist in what and how topics are discussed, and the content of the couples’ sexual health conversations. The synergistic nature of timing, motivation, and context appeared to be a determinant of sexual health conversation content and influenced subsequent interpersonal health-promotion behavior—which gives rise to the second thematic category: *Leveraging Conversations toward Sexual Health Promotion*. This study’s findings demonstrate an opportunity and the importance for sexual health conversations as an entry to one’s overall health.

### 4.1. Conversation Dimensions

Conversations among couples in this group of participants were either spontaneous or ongoing, but held intention. These conversations led to sexual health strategies such as the adoption of routinized HIV screening and efforts toward building intimacy. Understanding the dimension of timing is important for educational and engagement purposes of both health consumers and health providers. Knowing when sexual health conversations should incorporate certain information can have implications for when certain interventions (i.e., condom use, disease disclosure) can be introduced or reconsidered in a relationship. For example, routinized HIV testing during pregnancy may be an opportunity for inviting male partners to be screened for HIV infection [41,42]. Despite the demonstrated efficacy of couple-based approaches to engage the male partner in the antenatal period, these approaches in the U.S. are under-utilized and implementation needs are still unknown [43]. Similarly, regarding the timing and context of conversations, the impact of prescription drugs for metabolic conditions or mental health issues should not be underestimated on sexual functioning and subsequent relationship quality. Research and practice standards [43] support pre-emptive shared decision making and planning between health care providers and patients for improving patient outcomes in clinical settings. Pre-emptive planning in a shared decision process with health providers can support couples so that they can be prepared for sexual functioning issues, and have in place plans to address this issue. Such context can cause relationship discord as unresolved sexual functioning issues, which were a source of tension for a few couples in the study. Relatedly, metabolic issues in and of themselves can have an impact on sexual functioning [44], and can therefore be a source of tension within couples. This educational content should be discussed between partners, and undergird providers and patient communication. These conversations with providers should not be limited to side effects but include a plan of action for patients and their interpersonal relationships.

In this study, motivation for couples’ conversations were intrinsic and extrinsic, with both sources shown to hold equal weight in initiating conversations and subsequent goal-setting in adopting behavior. Some of our findings in this regard are similar to another qualitative study which explored long-distance couples’ discussions of relationship boundaries [45]. In that study, couples reported initiating sexual health conversations throughout the relationship for various reasons (e.g., defining the relationship, sexual exploration, and wanting to get to know each other more). McRae and Cobbs (2020) reported three types of motivation for relationship discussions among couples. Among their sample, motivations for these conversations were identified as either approach (e.g., clarifying nature of relationship, relationship maintenance, sexual exploration, and getting to know each other), avoidance (e.g., managing jealousy/insecurity, avoiding distress, and limiting extra-dyadic interactions), or ambiguous goals (e.g., just to have a discussion). The contexts of these conversations were also explored and showed some similarities across the McRae and Cobbs (2020) sample and the current study sample. Participants in the study by McCrae and Cobb (2020) were more concerned about having these conversations for individual or relationship gain. In our study, these conversations largely varied in context and pertained to sexual health, engagement in or considerations of sexual health behaviors, and relationship development or enhancement. The nuances in findings between the two studies may be attributed to our sample, having nearly one-third of couples where one or both partners living with HIV infection, being older (average age 43 vs. 24.5), Black-identified compared with a majority White and East Asian, and not long-distance couples. The difference in findings may also be attributable to our study purpose and the particular prompt that initiated discussion among our sample. Despite these differences, the motivation and intentions of couple’s conversations are important to recognize and analyze for leverage for intervention development.

### 4.2. Leveraging Conversations toward Sexual Health Promotion

An important consequence of sexual health communication is effective in goal setting and the adoption of health behaviors. There is a potential for not all communication to be effective which may result in discord or unresolved issues, whereas other communication can build relationship integrity. One meta-analysis found that issues of sexual functioning evoked different patterns of conversation based on the dimensions of the sexual health functioning issue that was being addressed. More importantly they found that the quality of the communication had bearing on sexual functioning [46]. We did not measure communication quality or effectiveness in this current study; however, couples reported on leveraging sexual health conversations toward health-promotion behaviors, which became the second thematic category. For the couples in this sample, there was evidence that some sexual health conversations led to the adoption of sexual-health-promoting behaviors such as PrEP considerations, routine HIV/STI screening, as well as strategies to enhance relationship health. However, in some instances, it appeared that certain conversations or communication did not consistently pre-emptively lead to goal setting or a plan of action, for example, in instances where an STI or HIV infection was detected in a partner or when condoms were dispensed. Such instances point to a need for couple/partner-centered education and intervention to assist certain couples in more directed conversation and joint goal setting. This was also reflected in the quantitative survey findings where couples indicated that certain behaviors were an individual decision and not a joint one. Research also notes that gender differences in communication and norms may impact engagement in sexual health behaviors such as condom use [47]. In our study, we did not note sex differences in communication that impacted discussion.

Among this sample, for all types of sexual activities, condoms were rarely used. This is not uncommon for couples, regardless of their HIV serostatus, [48] and hence perhaps should not be expected in long-established couples. Research has indicated that couples building intimacy will dispense of condoms as a symbolic gesture of trust [49,50], as was articulated with at least one couple in this study. Furthermore, introducing condom use after sex has commenced may be perceived as a lack of trust in the partner; therefore, couples may not discuss condoms [51] or other related topics such as HIV screening. However, research indicates that there is relational benefit to discussing sexual behavior and preferences as part of establishing relationship intention [52], including condom use. The implications here for health consumers, such as self-defined couples, and HIV/STI/sexual health providers alike, is that these conversations are an opportunity to introduce explicit self-directed interventions such as sexual or relationship agreements to influence subsequent behaviors [53]. Agreements ensure that there is mutual understanding, explicit goal setting, and contingency plans should there be a breach in relationship expectations. The creation of sexual or relationship agreements is an opportunity for couples and other intimate partners that health providers can introduce patients and their partners. In these agreements, providers can communicate and educate about biomedical interventions such as PrEP and PEP, and introduce behavioral interventions such as routinized HIV/STI screening. It can also be used to promote conversations regarding the effectiveness of antiretroviral treatment that ensures optimal viral suppression. Healthcare providers could also leverage agreements to have conversations about other sexual functioning issues or pleasure that may influence either relationship integrity or adverse sexual health outcomes.

### 4.3. Implications for Health Promotion

Having insight to the dimensions of sexual health conversations and subsequent behaviors has implication for healthcare and health providers to be cognizant of support needed for couples/partners including specific language/messaging to engage patients. For example, messaging of certain key concepts, such as undetectable equals untransmittable (U=U) in the context of HIV infection, is important for sexual and relationship health. IT is also important for people living with HIV or people wanting to partner (or conceive) with persons living with HIV infection [54]. To be armed with the knowledge that HIV, and other STIs can be effectively treated, and treatment has evolved to mitigate transmission, allows for the development for healthy conversations and sexual relationships among health consumers. The incorporation of intimate partners in clinical and community settings for sexual health promotion is crucial in stemming HIV/STI transmission and enhancing sexual health and wellness. Additionally, appreciating such diversity in couples’ experiences can provide insights to where interventions can leverage what couples already do or talk about to enhance their sexual, relationship and overall health.

Knowing that couples in this sample, especially male partners, engaged in routinized HIV screening as a disease preventative strategy, can be leveraged in community- and clinic-based settings via intervention in these spaces. Research has shown the acceptability of home-based testing and peer-based programming for optimizing HIV/STI testing among male persons [55,56] due to increased privacy, comfort, and autonomy. Optimizing existing behaviors among this group may address the fact that in the U.S., current HIV prevention interventions have grossly neglected the needs and realities of certain heterosexual persons who maintain a persistently high level of susceptibility to HIV/STIs [57,58], such as Black heterosexual/bisexual males and their female partners. Despite this possibility, there was less discussion among the current sample on how couples would handle a positive HIV/STI test result. Additionally, although routine HIV screening in itself is a sexual-health-promoting activity, when conducted in the context of infidelity or distrust within the relationship, this may impose limitations on the benefit because the underlying issue is not addressed (i.e., relationship commitment or intention).

### 4.4. Considerations for Future Research

Couple-centered approaches in clinical and community-based settings for Black couples are warranted to address ongoing HIV/STI transmission, vulnerability, functioning issues, and to strengthen healthy relationships. Quantitative results showed that couples may not have reconciled expectations for extra-dyadic behavior or believe that the only behavior that can be discussed or “controlled” is what occurs within the relationship. This is of significance because concurrent partnerships, and not number of sexual partners is the one unique contributor to heightened HIV/STI susceptibility among Black persons [59,60,61,62]. Specifically, concurrent non-consensual non-monogamous relationships contribute to heighten HIV susceptibility [61]. In light of the findings here, whereby communication about extra-dyadic behavior and relationships appear to be a source of tension among some couples, future research directions should include broadening of what we mean by couple among people categorized as heterosexual. For some persons and populations, the concept of couples require expansion in order to characterize intimate partners who may not be married or committed in traditional ways, but in which a significant level of investment across partners may exist. Couple-based research and other partner analysis reports that for some sub-populations, persons may have more than one main partner and that these partners may have equal or high significance in their sexual and social lives [57,63,64]. These partners may collectively compliment different individual attributes of a person. Therefore, future research is needed especially for Black persons regarding consensual non-monogamy (vs. infidelity) and the tailoring of sexual health promotion strategies for this populations given its unique contribution to health disparity among Black persons [62,65,66]. Especially given the experiences and accommodations Black heterosexual couples may make to maintain relationships and cope with sex-based and anti-Black stigma [19,67].

This current report revealed that couples are having sexual health conversations, which have resulted in subsequent adoption of sexual-health-promoting behavior. Such attributes and sexual health conversation indicate opportunities for intervention development that can leverage couples’ strengths in disease prevention, sexual health or relationship health more broadly. Future research could also explore factors of sexuality related to ones’ experiences due to their social standing and the impact of sex-based and anti-Black stigma on relationship integrity. One study demonstrated that negative partner-affective reactivity to daily experiences of racism among partners was associated with lower relationship quality in Black couples [28]. Another longitudinal study with Black-identified heterosexual married couples showed that depression predicted low marital satisfaction among male partners [68]. The associations between depression and relationship satisfaction among men is known [67]; however, the broader implications may not be as well studied among Black couples. Such experiences such as various social stigmas are known to engender depression and other mood disorders which has been associated with engaging in extra-dyadic behaviors and sexual behaviors that heighten one’s vulnerability to relationship discord and adverse sexual health outcomes [66]. Future research should also incorporate these factors given the role of anti-Black and anti-sexuality stigmas impact on mood disorders and relationship satisfaction.

Research continues to show that disclosure of HIV and STIs is difficult, and a lack of disclosure could result in delayed treatment and sustained community prevalence [68,69]. Research also demonstrates that assistance with disclosure via partner/couple-centered HIV/STI counseling and testing is effective in preventing transmission and sustaining people in HIV care [70,71,72,73]. Despite this, implementation of couple-centered interventions targeted towards Black couples such as EBAN, which has proven to be a culturally relevant couple-based intervention that promotes relationship and sexual health for Black/heterosexual couples [74], or Testing Together [75], which has demonstrated efficacy in joint HIV screening, HIV status disclosure and prevention of transmission for couples are lagging in U.S. settings. Other studies have also suggested a need for interventions that incorporate daily experiences of racism due to its interpersonal influence among Black couples and relationship health [28]. Expedited partner therapy (EPT) which has just been expanded in New York State is an opportunity for more flexibility in ensuring that partners are treated for STIs [76] and to introduce other strategies such as PrEP/PEP. However, it still requires the index partner to disclose their STI diagnosis and any related behavior to their partner without assistance with communication. Therefore, a need remains in implementing interventions, which engender sexual health communication and behaviors. Future research can include further assessments and implementation of engaging male persons who have relationships with women to have routinized access to home-based HIV/STI screening.

### 4.5. Limitations

A strength of this study is the use of an asset-based lens to describe the experiences of Black-identified couples. Despite the significance of this study’s findings, there are some limitations. Studies on Black couples have suggested that experiences of societal racism can greatly impact relationship integrity [58,77] and that these experiences and contexts can engender discord further disallowing couples’ communication regarding sexual health. Therefore, one potential limitation was the lack of a psycho-social assessment in the sexual health survey. A greater variation in the age of couples in tandem with length of relationship may have been warranted given that most of the couples had been together for over 5 years and most were older than 30 years old. This resulted in couples who may have a bit more lived experienced in cultivating relationships that can engender subsequent conversations regarding sexual health. Findings here may juxtapose a sample of couples who are developmentally younger, who may be navigating initiating sexual health conversations, and who may not think about their health external to themselves. Additionally, though we do not endorse single analytical approaches as a limitation in studies because the research drives the question, this pilot study was heavily qualitative and the quantitative application utilized was for descriptive purposes, not necessarily for inferencing. This allows for a sample size that is common for this type of study. Therefore, further research can utilize a more mixed methodological approach to quantitatively and qualitatively contextualize these conversations, perspectives and behaviors.

## 5. Conclusions

The occurrence of sexual-health-promoting behaviors among the couples are an asset and an opportunity to leverage a feature of the relationship to maintain sexual health or enhance it. Couple’s reports in this study undergirded the fact communication of desires and boundaries is a sexual-health-promoting activity. This finding aligns with similar studies conducted across different population of couples that demonstrate the importance of sexual health communication in relationship and overall health [26,53]. The findings of this study are of significance, given the persistent health disparities in HIV/STIs experienced by Black persons.

Current research is limited in the use of an asset-based lens when characterizing the sexuality of Black persons. In fact, one systematic review found that of the approximately 250 articles about Black women’s sexualities, only 6.5% engaged in a sex-positivity approach [78]. Although acknowledging threats to sexual health is important, exploring the components of people’s sexual lives that promote health are equally significant. In this study, sexual health conversations were ongoing, varied in context, content and motivation, and represented efforts towards sexual health promotion. These conversations and communication also proved to be part of a larger and more general health promoting strategy for couples. Findings in this study demonstrated that such interpersonal conversations are assets even when there is tension whereby couples are able to mitigate issues, and work towards joint sexual health goals. Understanding attributes of sexual health conversations has several implications for intimate partners. It can normalize conversations of sensitive subject matters, of conversations being diverse in how they occur and potentially addresses challenges and subsequent stigma regarding sex, sexuality, and sexual health and wellness. Normalizing these conversations can facilitate decision making and ease adoption of sexual health and wellness strategy as an integral part of overall well-being. Findings in this study align with calls for sexual health as a U.S. national priority [2] and the adoption of an ecologically targeted, asset-oriented sexual health framework as way of normalizing sexual health conversations.

## Figures and Tables

**Table 1:** Sample characteristics (N = 56).

Sample Size	Individual *n* = 56 (%)	Couple-Level *n* = 28	
		Partners Concur or Have Same Characteristic ^a^	Partners Do Not Concur or Differ on Characteristic ^a^
Demographic Characteristic	n (%)	N (%)	N (%)
**New York State Metro area**		28 (100)	0 (0)
**Buffalo**	20 (36)		
**New York City**	14 (25)		
**Rochester**	22 (39)		
**Racial ethnic background**		18 (64)	10 (36)
**Black American**	40 (71)		
**Black Hispanic/Afro-Latin**	8 (14)		
**Other Black identity**	8 (15)		
**Reported sex assignment**		-	-
**Male**	28 (50)		
**Female**	28 (50)		
**Current gender identity**		-	-
**Man**	28 (50)		
**Woman**	28 (50)		
**Current sexual identity**		24 (86)	4 (14)
**Heterosexual**	50 (89)		
**Other identity/Don’t do labels (no label)**	6 (11)		
**Education attainment**		14 (50)	14 (50)
**Some high school (did not complete)**	11 (20)		
**High school diploma or G.E.D.**	21 (38)		
**Training program or Associate’s degree**	13 (23)		
**College graduate or higher degree**	11 (19)		
**Employment status**		19 (68)	9 (32)
**Full-time**	19 (34)		
**Part-time**	9 (16)		
**Unemployed**	8 (14)		
**Disabled/Student/Part-time**	20 (25)		
**Relationship type**		21 (75)	7 (25)
**Married, monogamous**	18 (32)		
**Engaged, living together**	7 (13)		
**Girlfriend/Boyfriend**	27 (55)		
**Other**	4 (7)		
**Relationship length**		21 (75)	7 (25)
**Less than 5 years**	19 (34)		
**5 years or more**	37 (66)		

Notes: ^a^ For each couple, both partners responses to the same question were compared to assess whether they concurred or not about their metro area residence, racial ethnic background, current sexual identity, education attainment, employment status, relationship type, and relationship length.

**Table 2:** Relationship dynamics of the sample at the individual and couple level.

	Individual Mean	Average Absolute Score Difference
**Sample size**	N = 56	N = 28
**Relationship dynamic**	*M* (SD)	*M* (SD)
**Preferences for sexual health outcomes [range: 1–5] ^a^**		
**STI/HIV prevention**	4.30 (1.31)	1.18 (1.45)
**Using condoms with main partner**	4.36 (1.26)	0.86 (1.23)
**Having sex with someone other than your main partner**	3.41 (1.85)	1.89 (1.75)
**Whether you will use condoms when having sex with someone other than your main partner**	3.50 (1.91)	1.43 (1.84)
**Getting an STI or HIV test**	3.50 (1.79)	1.36 (1.55)
**Communal coping on communication to reduce HIV threat** **scale [range: 1–5] ^b^**		
**Using condoms when we have sex with each other**	2.95 (1.68)	1.25 (1.42)
**Limiting the number of other sex partners**	3.48 (1.73)	2.04 (1.54)
**Deciding about either of us having sex outside our relationship**	2.75 (1.79)	2.36 (1.60)
**Using condoms when either of us has sex outside our relationship**	3.14 (1.80)	1.71 (1.64)
**Getting tested regularly for STDs and/or HIV**	3.73 (1.51)	1.25 (1.37)
**Being sexually faithful to each other**	4.25 (1.06)	0.79 (0.95)
**Sexual relationship power scale**		
**Relationship control [range: 1–4] ^c^**	1.76 (0.51)	0.60 (0.47)
**Decision making dominance [range: 1–3] ^d^**	1.94 (0.32)	--

Notes: ^a^ Response options ranged from *‘Your issue/decision = 1 to ‘Our issue/decision’ = 5* regarding attitude on whether the partner thinks the topic is an individual versus collectively a joint issue/decision. ^b^ Response options ranged from *‘Not to any extent at all’ = 1* to *‘To a great extent’ = 5* about communicating on such topics with one another. ^c^ Response options ranged from *‘Strongly disagree’ = 1* to *‘Strongly agree’ = 4*, with the statement asking about individual perception about this aspect of their relationship. ^d^ Response options were *‘Your partner’ = 1, ‘Both of you equally’ = 2,* and *‘You’ = 3.*

## Data Availability

With the appropriate data use agreement data between institutions de-identified results from this study can be made available.
